# Folding Properties of Cytosine Monophosphate Kinase from *E. coli* Indicate Stabilization through an Additional Insert in the NMP Binding Domain

**DOI:** 10.1371/journal.pone.0078384

**Published:** 2013-10-30

**Authors:** Thorsten Beitlich, Thorsten Lorenz, Jochen Reinstein

**Affiliations:** Department of Biomolecular Mechanisms, Max Planck Institute for Medical Research, Heidelberg, Germany; Instituto de Tecnologica Química e Biológica, UNL, Portugal

## Abstract

The globular 25 kDa protein cytosine monophosphate kinase (CMPK, **EC ID: 2.7.4.14**) from *E. coli* belongs to the family of nucleoside monophosphate (NMP) kinases (NMPK). Many proteins of this family share medium to high sequence and high structure similarity including the frequently found α/β topology. A unique feature of CMPK in the family of NMPKs is the positioning of a single *cis*-proline residue in the CORE-domain (*cis*-Pro124) in conjunction with a large insert in the NMP binding domain. This insert is not found in other well studied NMPKs such as AMPK or UMP/CMPK. We have analyzed the folding pathway of CMPK using time resolved tryptophan and FRET fluorescence as well as CD. Our results indicate that unfolding at high urea concentrations is governed by a single process, whereas refolding in low urea concentrations follows at least a three step process which we interpret as follows: Pro124 in the CORE-domain is in *cis* in the native state (N^c^) and equilibrates with its *trans*-isomer in the unfolded state (U^c^ - U^t^). Under refolding conditions, at least the U^t^ species and possibly also the U^c^ species undergo a fast initial collapse to form intermediates with significant amount of secondary structure, from which the *trans*-Pro124 fraction folds to the native state with a 100-fold lower rate constant than the *cis*-Pro124 species. CMPK thus differs from homologous NMP kinases like UMP/CMP kinase or AMP kinase, where folding intermediates show much lower content of secondary structure. Importantly also unfolding is up to 100-fold faster compared to CMPK. We therefore propose that the stabilizing effect of the long NMP-domain insert in conjunction with a subtle twist in the positioning of a single *cis*-Pro residue allows for substantial stabilization compared to other NMP kinases with α/β topology.

## Introduction

Since the ground breaking discovery by Anfinsen *et al.* [1] that proteins indeed self-assemble to the native state, the correlation of primary structure (amino acid sequence) and the folding mechanisms of proteins has been subject of intense research in order to understand the basic underlying principles of protein folding [2], [3]. Since then substantial insight has been gained in the folding of many small proteins with a size of up to around 100 amino acids. Today computer-based folding simulations have reached a level where predictions of experimentally relevant timescales (seconds) of small proteins (∼100 amino acids) give satisfying results [4].

With the increasing amount of new biophysical techniques and detection systems, research now also focuses on the folding of proteins in the range of 200 amino acids and even above [5]. While small proteins with less than 100 amino acids often fold through a two-state transition, larger molecules usually show more complex folding kinetics. For these proteins, on- and off-pathway intermediates as well as heterogenic protein species, e.g. due to formation of disulfide bridges or proline isomerization, are part of the folding landscape [3], [6]. The observed intermediates often display significant amount of secondary structure but no well-defined global tertiary structure. These species are often prone to aggregation due to exposure of hydrophobic stretches, and are thus partially involved in numerous pathologies and human neurological disorders like Alzheimer’s disease and others [7]. Therefore knowledge about folding pathways and specifically the characteristics of the intermediate structures involved are essential to understand aggregation processes of proteins [3], [8].

One important approach to gain such knowledge is the comparison of folding properties in protein families, specifically with similar topology, yet not necessarily high sequence similarity or identity [5], [9]–[11].

Proteins from the NMPK family are highly suitable targets for such an approach since they are moderately sized with about 20–27 kDa, are mostly monomeric and accessible with many biophysical methods including NMR due to their high solubility. NMP kinases are found in all organisms and play a key role in the cell metabolism. In bacteria, phosphorylation of each nucleotide is achieved by its distinct kinase with high specificity for the appropriate substrate [12]. Due to their central role in anabolic nucleotide phosphorylation, NMP kinases are of special interest in pharmaceutical drug design for antiviral and anticancer as well as malaria therapies [13]–[16], where they are used in activation of nucleoside analog prodrugs like acyclic nucleoside phosphates [17]. The best characterized member of the NMP kinase family is AMP kinase [14], [18], including many structures from several organisms and in different states [19]–[21]. In addition, the structures of several other members of the NMP kinase family were determined e.g. UMP/CMP kinase from *D. discoideum* [22] (UMPK), yeast TMP kinase [13] and mouse GMP kinase [23].

**Figure 1:**
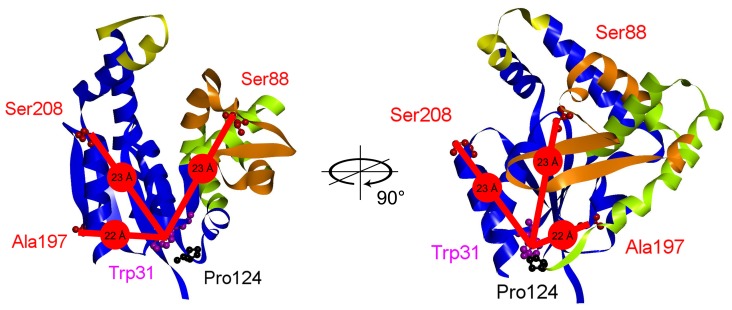
Structure of CMP kinase. Ribbon diagrams of CMPK crystal structure (**PDB ID: 2CMK**). The relevant amino acid residues used in this work are displayed as balls and sticks and labeled in individual colors. The intramolecular distances between Trp31 (C3a) and the amino acid (Cβ) used for addition of the fluorescent label AEDANS are indicated in red. Yellow: LID-domain (Leu160– Glu172), green: NMP-domain (Gly37 - Leu113), blue: CORE-domain. The long insert specific for CMPK (Leu63– Gln102) is colored in orange.

The 25 kDa protein CMP kinase from *E. coli* (CMPK) also belongs to this family and its structure alone and in complex with CDP was solved by Briozzo *et al*. [12] and classified as α/β-protein, including a P-loop motif, which is typical for these phosphoryl group transferring enzymes [24]. Like other members of this family, the protein consists of three domains (Fig. 1): the CORE domain, which contains the central five-stranded β-sheet and several surrounding α-helices, the LID-domain, which covers the phosphate binding pocket, and the NMP-binding-domain, which binds the specific NMPs [25]. The LID- and NMP-binding-domains are highly flexible in structure and undergo large conformational changes during the catalytic cycle with an induced-fit mechanism triggered by substrate binding to prevent unproductive hydrolysis of ATP [26].

**Figure 2:**
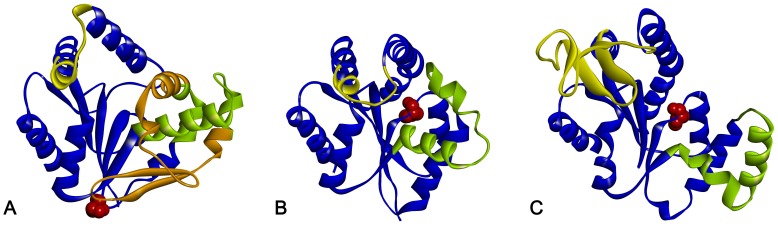
Structural comparison of different NMP kinases. Ribbon diagrams of CMPK (a), UMPK (b) and AMPK (c). For comparison, the individual domains are consistently colored: CORE-domain (blue), NMP-domain (green), LID-domain (yellow). The long insert of CMPK (orange) significantly increases the surface between CORE and NMP-domain. The single *cis*-proline of each protein is colored in red. While the position of this residue within a flexible region is conserved between UMPK and AMPK, it is shifted to a hinge-region in case of CMPK.

Unique for CMPK from *E. coli* is an insert of 40 residues in the NMP-binding-domain. In contrast, AMP kinase from *E. coli* (AMPK) has a large insert in the LID domain, whereas UMPK does not contain inserts in the LID or NMP-binding domains. Shortly after this insert at the end of the NMP-binding domain follows the single *cis*-proline residue Pro124 separated by one additional α-helix. This positioning is unique among NMP kinases since most of them also contain a single *cis*-Pro residue after the NMP-binding domain, but separated by an α-helix and an additional β-strand (Fig. 2).

We have thus analyzed the equilibrium and kinetic parameters of CMPK folding by a variety of spectroscopic methods to assess how these two changes in topology affect folding in comparison to other NMP kinases. In particular, AEDANS probes, attached to different positions, served as Förster resonance energy transfer (FRET) acceptors in combination with the single tryptophan residue Trp31 as FRET donor (see Fig. 1). In the native state, the endogenous Trp31 of CMPK is located at a surface exposed cavity that is formed by the N-terminal subdomain of the CORE-domain and Pro124. Our data suggest that folding of CMPK is highly concerted and involves at least one intermediate state with considerable secondary structure as opposed to UMPK where most secondary structure is only formed upon reaching the native state. Even more striking is the high kinetic stabilization that slows down unfolding around 100-fold compared to other NMPKs.

## Results

### CMPK Variants for FRET Labeling are all Catalytically Active

Expression and purification of wild type CMPK with attached His-tag yields highly concentrated and pure protein (see materials and methods) which is monomeric even at low salt concentrations as judged by analytical gel filtration. For the FRET-experiments described below several constructs were generated. To prevent double-labeling, the naturally occurring cysteine Cys22 was exchanged for a serine (C22S). In combination with the attached hexa-histidine tag these constructs are noted with an asterisk (*). For site-specific attachment of AEDANS, single solvent exposed amino acids (Ser88, Ala197, Ser208) located in different regions of the protein at an approximate distance of 22 Å to Trp31 in the folded conformation were additionally exchanged for a cysteine (see Fig. 1). The notation here is then *88, *197 and *208 respectively. Constructs carrying an AEDANS at the indicated position will be called (A+), the unlabeled proteins will be called (A−). For control measurements constructs without the single tryptophan as FRET donor were generated (W31F) and will be referred to as (D−). All generated constructs were catalytically active with activities ranging between 28 and 190% of wild-type activity. We assume that the overall sensitivity of catalytic activity to these amino acid residue exchanges remote from the active site originates from the highly dynamic induced fit mechanism of the enzyme.

### Equilibrium Urea Unfolding Shows One Transition

**Figure 3:**
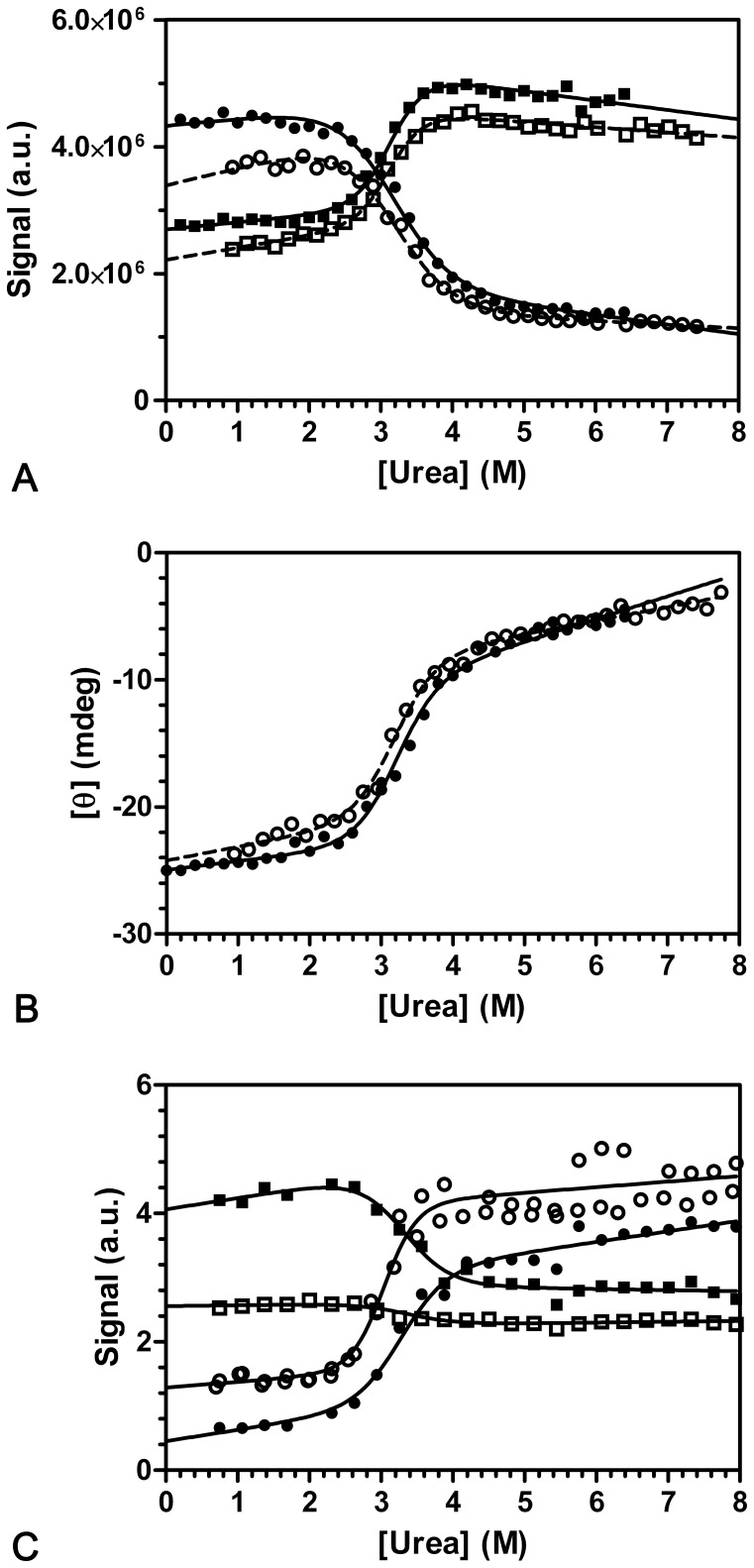
Urea induced unfolding followed by tryptophan fluorescence and CD. Unfolding/refolding transitions were recorded starting with initially folded (0.6 M urea, filled symbols) and unfolded (6.0 M urea, open symbols) CMPK. Tryptophan fluorescence was recorded between 305 and 500 nm. (a) displays the fluorescence intensities between 310 and 319 nm (•) as well as between 350 and 359 nm (▪). Urea dependence of CD at 222 nm is displayed in (b). Raw data of CD and fluorescence intensity was globally fitted to a two state transition, according to Santoro and Bolen [51]. The fits are displayed as solid (initially folded CMPK) and dashed (initially unfolded CMPK) lines (see text). (c) Equilibrium unfolding of *88 mutants. Tryptophan fluorescence (•, intensity at 370 nm) and AEDANS fluorescence(▪, intensity at 470 nm) with excitation at 296 nm. Open symbols indicate single labeled mutants (○, (D+A−); □, (D−A+)), the double labeled mutant (D+A+) is depicted by filled symbols (•▪).

For investigation of the thermodynamic stability of the protein, the intrinsic spectroscopic properties of CMPK were analyzed, using either the fluorescence signal of the single tryptophan (Trp31) (Fig. 3a) or the α-helical far-UV CD signal at 222 nm (Fig. 3b). Analysis of traces at individual wavelengths and separately for folding and unfolding indicates that results converge within given STD for the fitted values of m and D50 indicating that a two step transition is sufficient to describe the experimental data. We therefore relied on global analysis that gives fitted parameters with lower STD due to increased number of data points.

Since the same transition was observed for the refolding of CMPK after unfolding for 60 minutes in 6 M urea, the unfolding process was considered reversible (Fig. 3a/b, open symbols). Global analysis with a two-state transition model (Methods) gives a midpoint of transition at 3.2 M urea both with fluorescence and the CD measurements. The Gibbs free energy of unfolding (ΔG_u_) was calculated to 26.8 kJ·mol^−1^ with a cooperativity factor (m-value) of 8.39 kJ·M^−1^·mol^−1^.

To check for potential differences between the central CORE-domain carrying Trp31 and the NMP-domain, equilibrium unfolding was also analyzed for the CMPK *88 mutants (see below). In this case fluorescence of either Trp31 (measured between 320 and 400 nm) or the AEDANS fluorophore (measured between 430 and 550 nm nm) was used in a global fit anaylsis (Fig. 3c). All data support a reversible two-state transition model with a global midpoint of transition at 3.28±0.04 (D+A+), 3.04±0.03 (D+A−) and 3.37±0.2 (D−A+) M urea, andcorresponding m-values of 7.73±0.7, 10.8±1.1 and 7.3±3.0 kJ·M^−1^·mol^−1^.

### Folding Kinetics of CMPK – Definition of Rate Constants

**Figure 4:**
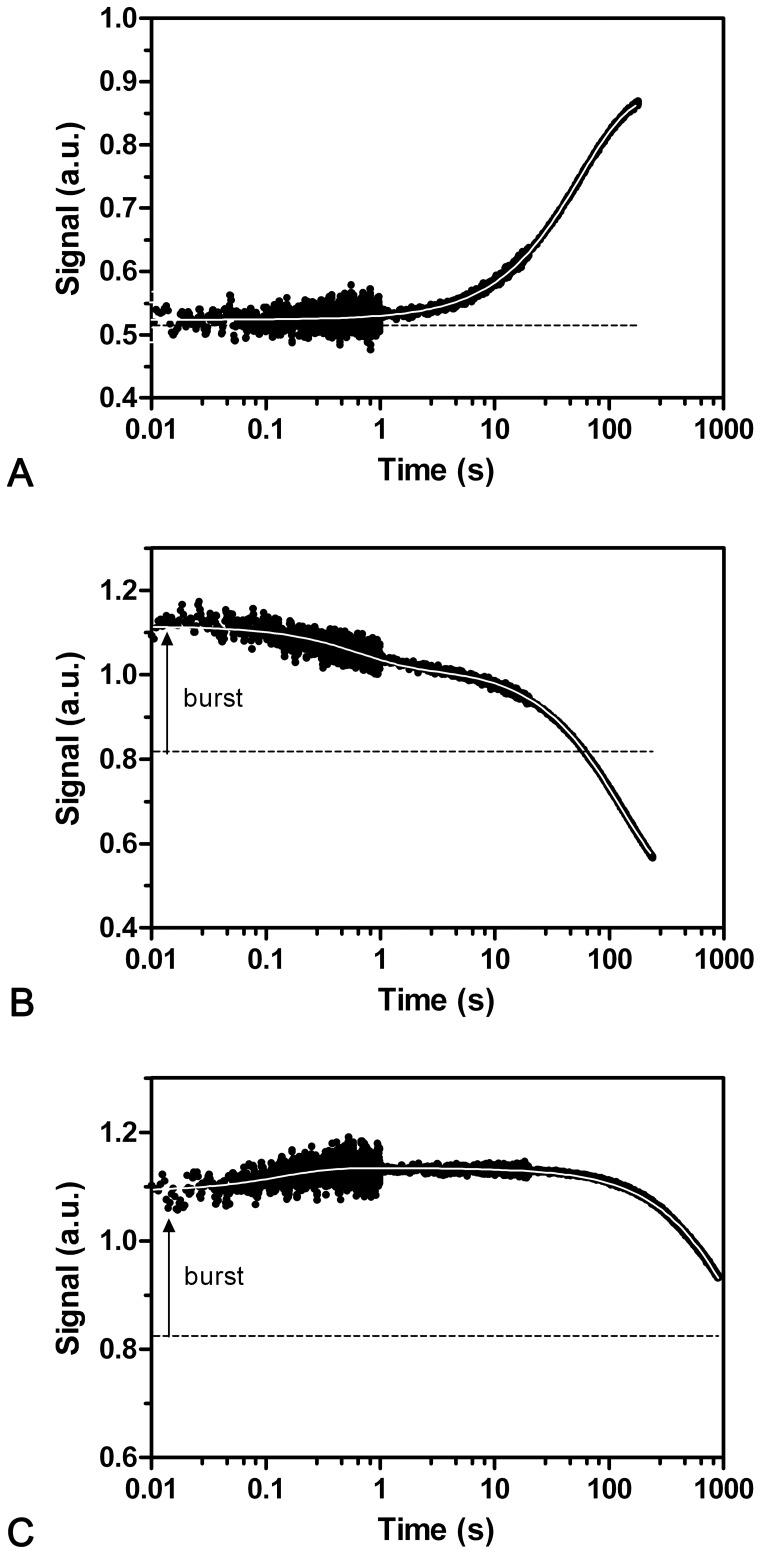
Unfolding and refolding kinetics of CMPK wt in the single-mixing stopped-flow setup. Upon excitation at 296(a), as well as the refolding transition from 6.0 to 0.9 (b) and 2.7 (c) M urea. The dashed lines indicate the urea dependent baseline signal of the folded (a) and unfolded (b, c) states determined from chevron analysis (see Fig. 4). All unfolding data can be fitted to a single exponential function with urea-dependent rate constant λ_U2(US)_ between 0.1 s^−1^ and 0.001. The refolding traces can be fitted to two exponentials ranging from 1 to 10 s^−1^ (λ_F1(RS)_) and 0.01 to 0.0002 s^−1^ (λ_F3(RS)_). Additionally, a burst phase can be observed in the refolding traces.

In order to investigate the kinetics of urea induced unfolding and refolding, a series of stopped-flow experiments were carried out. In a single mixing setup CMPK was either rapidly unfolded in urea concentrations above 3.2 M or refolded by dilution from 6 M urea into concentrations lower than 3.2 M urea. Whilst the unfolding kinetics of CMPK can be analyzed by a single exponential equation (Fig. 4a), the refolding kinetics show a burst-phase which can be deduced from the gain of the total amplitude (signal change within the dead time of the stopped-flow instrument of 3 ms) and two phases that can be kinetically resolved (Fig. 4b/c).

**Figure 5:**
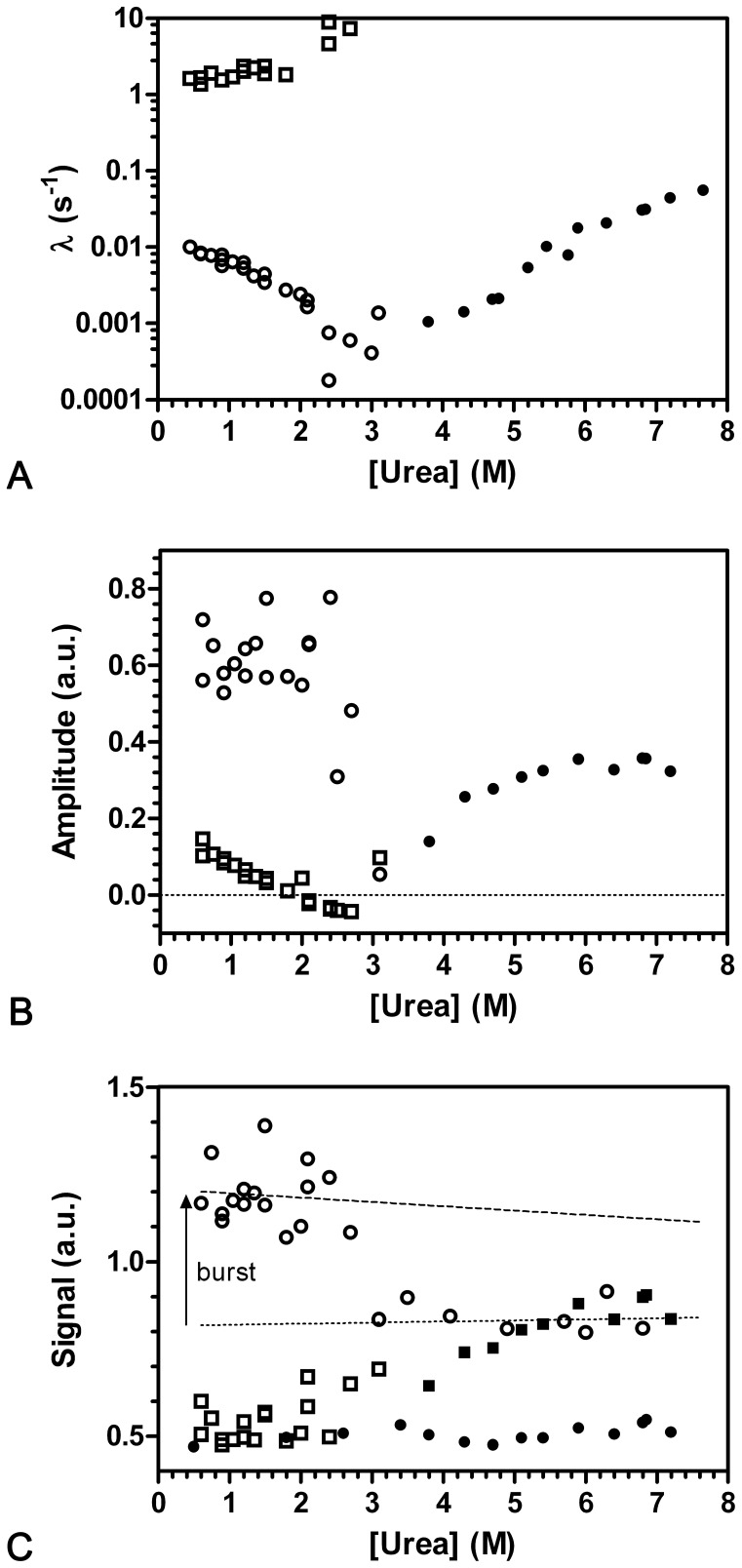
Un- and refolding kinetics of CMPK wt – Chevron Plot and end point analysis. Data was collected with a photomultiplier and 360(a) Apparent rate constants λ as a function of urea concentration. The slow unfolding (•) and refolding (○) transitions show a linear dependency in the semi-logarithmic plot. The fast refolding transition (□) shows an increasing rate constant towards intermediate urea concentrations and therefore suggests to be associated with an intermediate state. (b) Amplitudes of the observed kinetic traces. While the slow transitions show constant amplitudes (symbols like in (a)), the amplitude of the fast transition decreases and turns negative at 2.0 M urea. (c) End-point analysis of the unfolding and refolding transitions. Filled symbols represent unfolding transitions from 0.6 M urea, while open symbols depict refolding transitions from 6.0 M urea. Start (•) and end (▪) values are taken from extrapolation (t = 0 s and t = ∞ s) of the fit results (single exponential for unfolding, double exponential for refolding) of primary data (see Fig. 3). As guide to the eye, the dotted line indicates the extrapolation of signal intensity in the unfolded state. The dashed line represents the signal intensity of the first datapoints and therefore indicates the amplitude difference associated with the burst phase. The apparent differences in data quality concerning signal to noise ratio originate from different sampling times for individual time windows (see Methods).

To facilitate a consistent description of the data between different types of experiments, phases are consistently indexed according to the observed phases in double jump stopped-flow experiments as described below (fast: λ_F1(RS)_ to slow: λ_F3(RS)_, Fig. 5a). The symbol λ indicates an observed transition rate constant (as opposed to microscopic rate constants which we could not resolve unequivocally), while the index differentiates between the observed transition (F, folding; U, unfolding), its rank within the sequence of totally observed transitions (1 = fast; 2 = intermediate and 3 = slow) and the according experiment (RS, refolding single-jump; US, unfolding single-jump; IR, interrupted refolding; IU, interrupted unfolding). The calculated amplitudes are labeled accordingly, in this case A_F1(RS)_ and A_F3(RS)_. Capital lambdas (Λ) indicate the observed rate constants obtained from secondary data, in this case fits of amplitude plots resulting from double jump experiments.

### Unfolding/Refolding Kinetics show One and Two Kinetically Resolved Transitions Respectively

The unfolding of CMPK in urea concentrations above 3.8 M is characterized by a single unfolding phase whose apparent rate constant (λ_U3(US)_) increases exponentially (linearly in the semi-logarithmic plot, chevron plot) with increasing concentrations of urea. The corresponding amplitude (A_U3(US)_) accounts for the total signal change indicating that there is no burst-phase. The refolding kinetics of CMPK could be determined between 0.6 M and 2.7 M urea. The rate constant for the fast phase λ_F1(RS)_ is almost independent (1.3–2.3 s^−1^) of the denaturant concentration for urea concentrations below 2.0 M. An increase at higher urea concentrations to values around 8 s^−1^ can be observed. The slow phase λ_F3(RS)_ decreases with decreasing amounts of urea (0.01–0.002 s^−1^) between 0.6 and 2.0 M urea. Since rate constants in the range of 0.001–0.1 s^−1^ are indicative for Xaa-Pro bond isomerization processes [27], λ_F3(RS)_ is most likely linked to prolyl-bond isomerization. λ_F1(RS)_ deviates from the typical linear dependency on the denaturant concentration. This deviation (rollover) could suggest that an intermediate is present in the folding mechanism [28]. Especially the increase in λ_F1(RS)_ with urea concentration is unusual for refolding reactions. Similar observations have been made for UMPK with increases in λ_1_ and λ_2_ [29]. Both cases can be related to theoretical considerations by Wildegger and Kiefhaber on folding of lysozyme [30] who explain such behavior by the presence of a fast folding off-pathway intermediate that has to be unfolded before the next folding transition.

In conjunction with the chevron plot (Fig. 5a), the amplitude plot (Fig. 5b) reveals λ_F3(RS)_ as the main folding phase. Over the entire concentration range in the refolding experiments, A_F3(RS)_ stays almost constant with average amplitudes of 0.6 a.u. On the other hand A_F1(RS)_ is strongly dependent on the denaturant concentration. A_F1(RS)_ decreases between 0.6 and 2.7 M urea and the amplitude turns negative at 2.0 M urea. Interestingly, the change of amplitude A_F1(RS)_ coincides with the rollover of the according rate constant λ_F1(RS)_ observed in the chevron plot.

To detect a possible burst-phase in the folding or unfolding reaction of CMPK, the initial and final signals of the different measurements were plotted against the respective urea concentration [31]. A deviation of the initial kinetic values from the baseline of the according equilibrium values is an indication of a signal change within the dead-time of the stopped-flow (3–4 ms, depending on setup conditions). This deviation can be observed in the refolding process of CMPK (Fig. 5c), where initial refolding data differs from unfolded equilibrium data by a significant increase in signal amplitude. A possible explanation for such a fast process could be a rapid formation of a folding intermediate from which the native structure is formed.

### Secondary Structure is Mostly Formed in Burst Phase of Refolding but also in two Kinetically Resolved Phases

**Figure 6:**
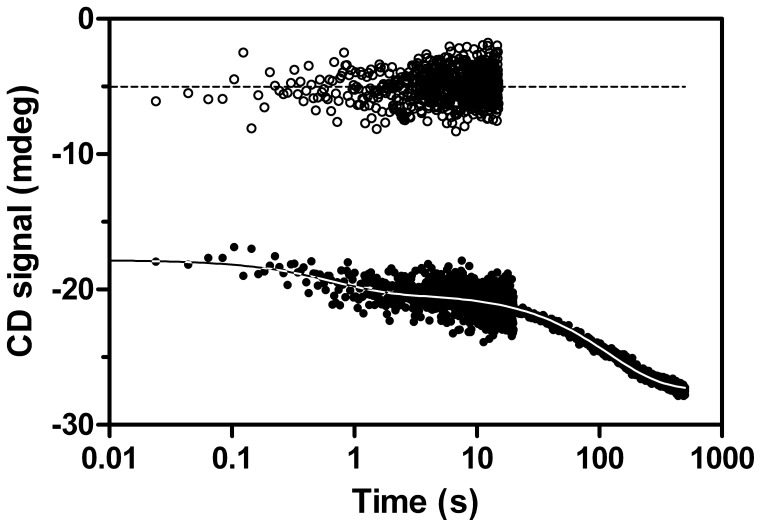
Sample of CMPK CD signal during refolding. In the unfolded state (open circles) some secondary structure elements remain. Within the burst phase, an immediate increase in CD signal corresponding to formation of secondary structure can be observed. Further on, refolding (filled circles) can be fitted to a double exponential function with rate constants in the range of λ_F1(RS)_ and λ_F3(RS)_. The apparent differences in data quality concerning signal to noise ratio originate from different sampling times for individual time windows (see Methods).

To address the question, in which of the observed folding phases appreciable secondary structure is formed, we followed refolding of CMPK by far-UV CD stopped-flow kinetics at 222 nm (Fig. 6). To this end, 100 µM CMPK was unfolded in 6 M urea for 60 minutes at RT prior to refolding by a tenfold dilution into buffer (0.6 M urea final). The kinetics for this reaction show a clear double exponential decay with rate constants of 2.8 s^−1^ and 0.0082 s^−1^, comprising 15% and 25% of the total amplitude. The remaining 60% are associated with an initial burst-phase. This is a very interesting observation, since it reveals that the majority of secondary structure is indeed formed within the first milliseconds of refolding, while the observed phases λ_F1(RS)_ and λ_F3(RS)_ contribute only to smaller amounts. Still the Xaa-Pro isomerization process associated with λ_F3(RS)_ affects secondary structure formation and therefore has a strong impact on the folding process of CMPK. Consistently, unfolding showed a single transition as observed with CD with a rate constant comparable to λ_U3(RS)_. Additionally, a burst phase comprising approximately 20% signal amplitude was observed. This indicates a fast unfolding of certain subdomains within the burst phase, while the majority of secondary structure is dissolved in the concerted slow unfolding transition.

### Interrupted Unfolding Reveals Additional Folding Phase

**Figure 7:**
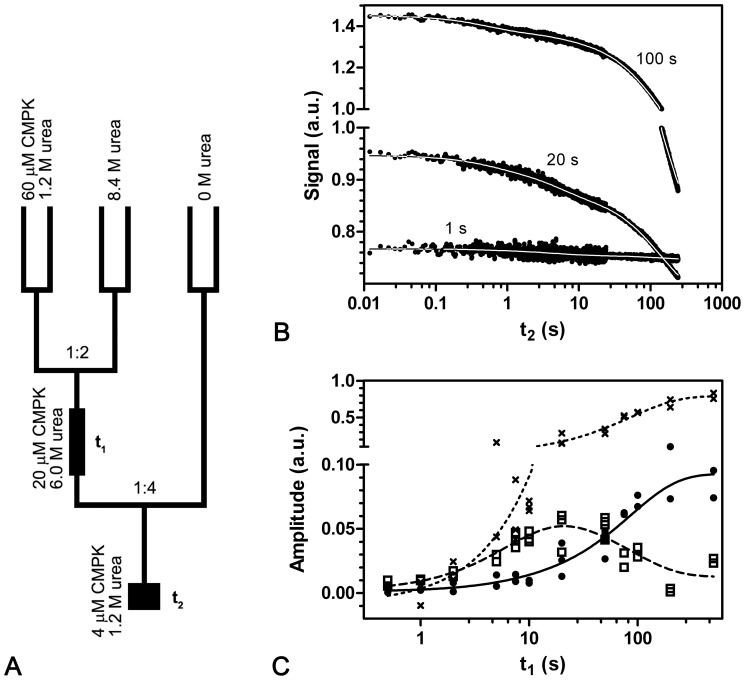
Interrupted unfolding of CMPK wt. The mixing-scheme of the interrupted unfolding reaction is displayed in (a). After unfolding in 6.0 M urea with incubation time t_1_, the protein is diluted into 1.2 M urea. The subsequent refolding is recorded as function of refolding time t_2_ (b). For short incubation times (<100 s), a transient intermediate refolding phase λ_F2(IU)_ can be observed. For long incubation times, the fast and slow refolding phases λ_F1(RS)_ and λ_F3(RS)_ described in the single jump experiments sufficiently describe the observed transitions λ_F1(IU)_ and λ_F3(IU)_. A secondary plot of the amplitudes A_F1(IU)_ (•), A_F2(IU)_ (□) and A_F3(IU)_ (×) corresponding to the rate constants λ_F1(IU)_, λ_F2(IU)_ and λ_F3(IU)_ is shown in (c). A global fit performed on these amplitudes yields rate constants of Λ_U2(IU)_ = 0.14 s^−1^ and Λ_U3(IU)_ = 0.012 s^−1^. The fits are depicted as solid (A_F1(IU)_), dashed (A_F2(IU)_) and dotted (A_F3(IU)_) lines. To account for the different scales in amplitude changes, the y-axis is splitted at 0.1 a.u.

To further investigate the proline *cis*-*trans* isomerization in the unfolded state, the double-mixing technique was used [27], [32]. In contrast to the single mixing experiments described above, two mixing steps were applied. In interrupted unfolding experiments CMPK was unfolded in a first mixing step with 6 M urea for various delay times t_1_ between 0.5–500 s. In a second mixing step, refolding was initiated by rapid dilution to a residual concentration of 1.2 M urea (Fig. 7a). For technical reasons including mixing ratios, concentration of urea stock solutions as well as comparability between interrupted un- and refolding experiments, 1.2 M urea had to be used as refolding concentration in double jump experiments. All kinetic traces were fitted globally to a triple exponential equation with shared rate constants of λ_F1(IU)_ = 2.0 s^−1^, λ_F2(IU)_ = 0.19 s^−1^ and λ_F3(IU)_ = 0.0068 s^−1^ for the different measurements (Fig. 7b).

A plot of the respective amplitudes versus the unfolding time (Fig. 7c) gives information about the accumulation of species during the unfolding reaction. For short unfolding times (<1 s) no refolding transition can be observed. For long incubation times, the refolding process (λ_F1(IU)_ and λ_F3(IU)_) is similar to the single-jump experiments described above with the two refolding phases λ_F1(RS)_ and λ_F3(RS)_. For comparison the observed rate constants of single and double jump experiments are listed in table 1. With intermediate unfolding periods (<100 s), a new refolding phase λ_F2(IU)_ is recorded, that is not observed for long incubation times.

**Table 1:** Observed rate constants during unfolding and refolding of CMPK wildtype.

	refolding 6.0 −>0.6		refolding 6.0 −>1.2					
	RS		RS		IU		IR	
λ_F1_ (s^−1^)	1.37	±0.09	2.36	±0.26	2.01	±0.03	5.88	±2.50
λ_F2_ (s^−1^)	–	–	–	–	0.187	±0.004	–	–
λ_F3_ (s^−1^)	0.00816	±0.00009	0.00628	±0.00006	0.00678	±0.00002	0.00464	±0.00043
	**unfolding 0.6 −>6.0**		**unfolding 1.2 −>6.0**					
	**US**				**IU**		**IR**	
λ_U1_ (s^−1^)	–	–			–	–	13.6	±0.2
λ_U2_ (s^−1^)	–	–			0.139	±0.227	–	–
λ_U3_ (s^−1^)	0.0180	±0.0005			0.0115	±0.0018	0.0145	±0.0001

The rate constants (λ) of the wildtype protein observed during unfolding (U) and refolding (F) are grouped according to time scales (indices 1–3) and experimental conditions (RS: refolding single-jump; US: unfolding single-jump; IU: interrupted unfolding; IR: interrupted refolding). Refolding was observed after unfolding in 6.0 M urea. For the unfolding experiments, CMPK was initially incubated in 0.6 M urea (US) or 1.2 M urea (IU, IR).

The intermediate phase λ_F2(IU)_ has a maximum amplitude A_F2(IU)_ at incubation times t_1_ of 20 seconds and decreases subsequently to values around 0 and is therefore not observed in the single-jump refolding experiment. The amplitudes A_F1(IU)_ and A_F3(IU)_ of the fast λ_F1(IU)_ and the slow λ_F3(IU)_ transition increase concurrently and show an average ratio of 7.6. The amplitudes A_F1(IU)_, A_F2(IU)_ and A_F3(IU)_ can be globally fitted to two exponentials as function of refolding time t_1_. This fit yields the two new secondary rate constants Λ_U2(IU)_ = 0.14 s^−1^ and Λ_U3(IU)_ = 0.012 s^−1^, where the capital Λ indicates that these transitions were obtained from secondary (Amplitude) data. The amplitudes A_F1(IU)_ and A_F3(IU)_ increase with Λ_U3(IU)_, while the intermediate process A_F2(IU)_ increases with Λ_U2(IU)_ and decreases with Λ_U3(IU)_.

Assuming that Λ_U3(IU)_ is associated with the proline isomerization step described for the unfolding reaction, A_F1(IU)_ and A_F3(IU)_ depend on the isomerization process and therefore belong to refolding from a *trans*-proline-state. A_F2(IU)_ on the other hand appears to be associated with the sub-species of partially unfolded CMPK containing *cis*-prolines. Interestingly, the process generating A_F2(IU)_ (with Λ_U2(IU)_) is not observed in the unfolding reaction, which indicates that a spectroscopically silent unfolding intermediate has to be involved. In order to yield the observed refolding transition after short unfolding times, this unfolding intermediate under refolding conditions would have to burst into a second intermediate with increased tryptophan-fluorescence, which could refold to the native state with the observed rate constant λ_F2(IU)_.

### Interrupted Refolding Confirms Folding Intermediate

**Figure 8:**
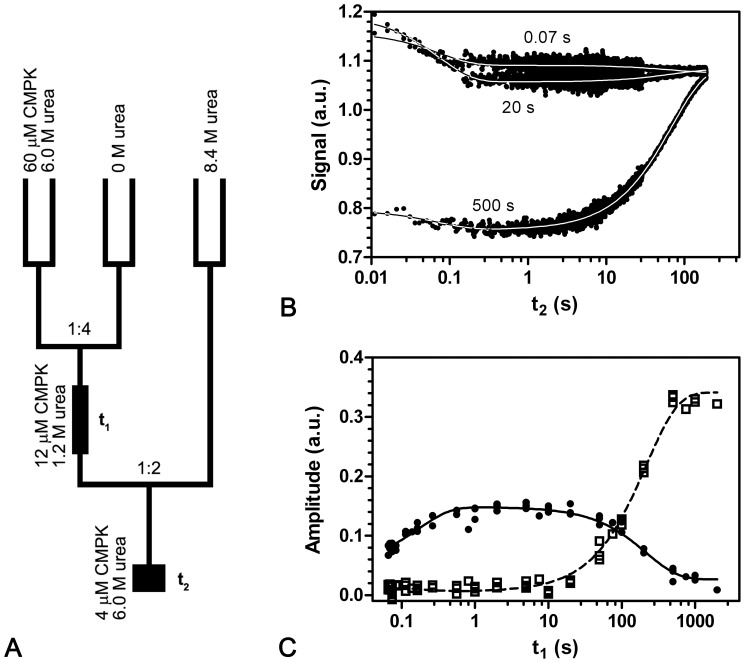
Interrupted refolding of CMPK wt. (a) Mixing scheme of the interrupted refolding reaction. After refolding in 1.2 M urea for incubation time t_1_, the protein is diluted back into 6.0 M urea. The subsequent unfolding is recorded as function of unfolding time t_2_ (b). For short incubation times (<100 s), a transient fast unfolding phase λ_U1(IR)_ can be observed. For long incubation times, the slow λ_U3(IR)_ unfolding phase also described in the single jump experiments appears. A secondary plot of the amplitudes A_U1(IR)_ (•) and A_U3(IR)_ (□) corresponding to the rate constants λ_U1(IR)_ and λ_U3(IR)_ is shown in (c). A global fit of this data yields secondary rate constants of Λ_F1(IR)_ = 5.9 s^−1^ and Λ_F3(IR)_ = 0.0046 s^−1^.

Since the native protein usually exhibits a higher activation energy towards unfolding than partially folded intermediates, the urea induced unfolding reaction of the fully folded structure should be slower compared to partially folded structures [33]. This characteristic can be used in an interrupted refolding experiment [34] to quantitatively monitor the formation of native molecules compared to intermediates. Unfolded CMPK (6 M urea for 60 minutes) was refolded in 1.2 M urea for 0.06–2000 s prior to a second unfolding step in 6 M urea (Fig. 8a). The unfolding kinetics could be globally fitted to a double exponential equation with shared rate constants of λ_U1(IR)_ = 13.6 s^−1^ and λ_U3(IR)_ = 0.015 s^−1^, respectively (Fig. 8b). The slower rate constant agrees well with λ_U3(US)_, whereas the fast one could not be determined with single jump experiments (see table 1). The amplitudes A_U1(IR)_ and A_U3(IR)_ as a function of refolding time t_1_ can be globally fitted to a double exponential with new rate constants Λ_F1(IR)_ = of 5.9 s^−1^ and Λ_F3(IR)_. = 0.0046 s^−1^ (Fig. 8c). A_U1(IR)_ (the fast unfolding process) increases with Λ_F1(IR)_ before it decreases again with Λ_F3(IR)_ and finally reaches a very low amplitude. This explains why this phase is not visible in single jump experiments. The amplitude A_U3(IR)_ on the other hand increases with Λ_F3(IR)_ to give a maximum amplitude that is twice the amplitude of the fast unfolding process. The slow secondary rate constant Λ_F3(IR)_ agrees well with the rate constant λ_F3(RS)_ observed in the single mixing refolding experiments.

Since the slow unfolding process is assumed to be associated with proline isomerization from *cis* to *trans*, the fast unfolding process λ_U1(IR)_ has to be associated with a CMPK configuration with Pro124 in the non-native *trans* conformation. Considering orientation and amplitude of this process, it could indeed describe unfolding from the fast folding intermediate (I^t^_2_) observed in the single-jump refolding reaction described above.

**Figure 9:**
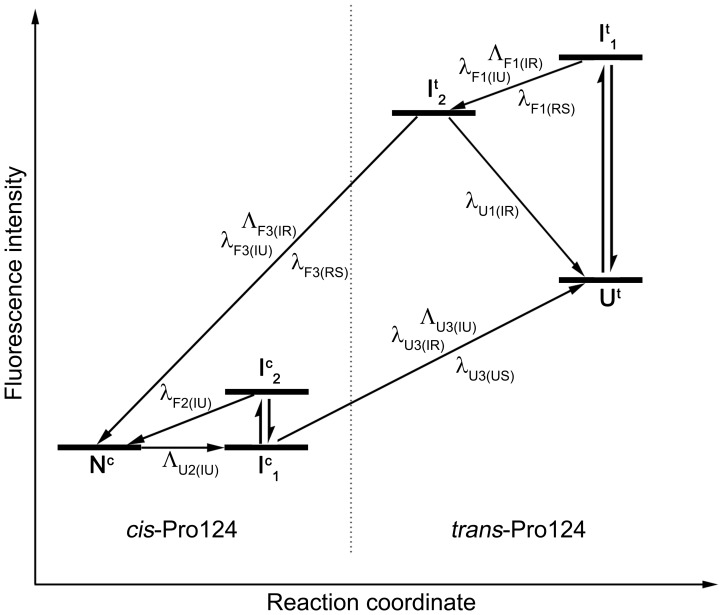
Working scheme for CMPK folding- and unfolding pathways. Folding scheme for folding and refolding of CMPK, omitting refolding from the U^c^ species. The x-axis represents the reaction coordinate, while the y-axis corresponds to fluorescence intensity of Trp31 during refolding of wildtype CMPK. Each horizontal line indicates a macroscopic species, as observed in the folding experiments. The native (N^c^) and unfolded state with trans-Pro124 (U^t^) are indicated separately. Vertical double arrows represent burst-phases. Arrows heading left indicate folding transitions, arrows heading right unfolding transitions. The transition rate constants corresponding to each arrow are positioned next to it. The dotted line indicates the energy barrier associated with the slow folding and unfolding process, separating the *cis-* from the *trans-* Pro124 species.

All results of the single and double jump experiments can be merged into a macroscopic folding scheme that describes the observed transitions (Fig. 9). In this scheme, the x-axis belongs to the reaction coordinate with the native state on the left and the unfolded state on the right. The y-axis represents the observed macroscopic fluorescence intensity. Transitions between different states are indicated by arrows heading left (folding) or right (unfolding), annotated with the associated observed rate constants. In this scheme refolding from unfolded proteins with *cis*-Pro124 configuration is not included. In general this species is difficult to characterize, since unfolding seems to be associated with *cis/trans* isomerization, so accumulation of the unfavored *cis* configuration cannot be easily accomplished. We therefore want to focus on the refolding transition from the *trans*-Pro124 species.

### Refolding with Peptidyl-prolyl Isomerases Shows Acceleration with Trigger Factor

**Figure 10:**
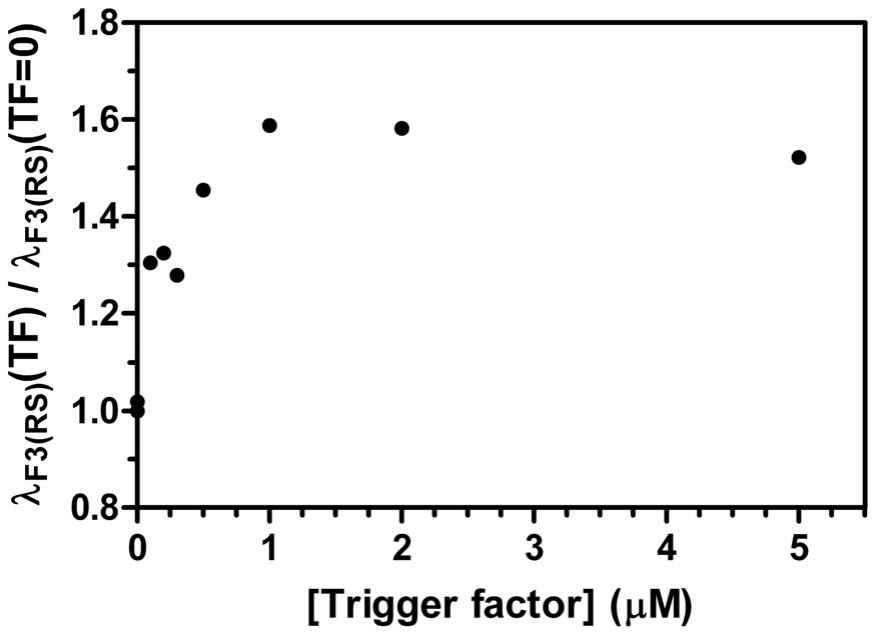
Acceleration of slow refolding phase λ_F3(RS)_ by *E. coli* trigger factor. The slow refolding phase λ_F3(RS)_ of 0.5 µM CMPK was analyzed at 360 nm during refolding in 0.6 M urea at different concentrations (0–5 µM) of *E. coli* trigger factor (TF). The ratio of λ_F3(RS)_(TF) to the refolding rate constant without trigger factor λ_F3(RS)_(TF = 0) is displayed as a function of TF concentration. Increasing concentrations of TF lead to an acceleration of λ_F3(RS)_. At a ratio of 2∶1 (1 µM TF) the ratio reaches a maximum of 1.6.

To assign the different phases in the refolding kinetics of CMPK to distinct folding processes, discrimination between parallel reactions either due to heterogeneity in the unfolded state or to the occurrence of folding intermediates has to be done. The heterogeneity of the unfolded state often results from different peptide bond isomers, in particular Xaa-Pro peptide bonds. Since our initial data suggest proline isomerization to be responsible for λ_F3_, we further scrutinized this hypothesis by an enzymatic assay. A direct test for a *cis*-*trans* isomerization process of a Xaa-Pro bond makes use of peptidyl-prolyl isomerases, specific enzymes that catalyze this type of reaction [35]. To that end we employed human cyclophilin A (Sigma), SlyD from *E.coli* and *E.coli* trigger factor (TF) to test for their activity on CMPK. Refolding was initiated by dilution of CMPK (unfolded in 6 M urea) into 0.6 M urea to a final concentration of 0.5 µM CMPK. In the presence of Cyclophilin A and SlyD, no acceleration was observed, whereas the presence of 0.2 µM TF led to a 1.3-fold acceleration of λ_F3(RS)_ as measured upon manual mixing in a fluorescence spectrometer (Fig. 10). Titration of TF into such a refolding assay of CMPK shows a linear increase in the observed refolding rate constants up to 1.6 fold at 1.0 µM TF where it levels out. This suggests that the slowest phase is connected to *cis*-*trans* isomerization of the Xaa-Pro bond at Pro124 and corresponding structural rearrangements. This small amount of catalytic increase in refolding rate could be explained by the amino acid Leu123 preceding Pro124, which leads to a decreased activity of trigger factor [36]. Furthermore secondary structure elements persisting in unfolded or intermediate conformations could block access to Pro124 and thereby prevent catalytic activity.

### Kinetic FRET Studies show Variations in Fast and Intermediate Refolding Phases

To further investigate structural changes during the folding process, the fluorescent dye AEDANS was attached at different key positions of the protein to serve as acceptor for FRET from excited Trp31 (see Fig. 1). The single tryptophan residue Trp31 is located in the first part of the CORE domain at the immediate border to the NMP domain (overall sequence of elements: CORE-NMP-CORE-LID-CORE [12]). It is located in a surface exposed hollow in close proximity to the single *cis*-proline residue Pro124. The positions of introduced cysteine residues Cys88, Cys197 or Cys208 are all in distance of 22–23 Å as calculated with the X-ray structure (**PDB ID: 2CMK**) [12] for C3aTrp31-CβCysNN distances (see Fig. 1) and are as follows.

Cys88 is located in the NMP-domain at the border of the 40 aa insert that is specific for CMPK. This position is comparable to amino acid 58 in AMPK where a label was introduced by Haas and co-workers [37] with the same purpose, that is to monitor movement of the NMP domain relative to the central core domain. Cys197 is located right before the last β-sheet that is still part of the CORE domain and thus expected to be fairly rigid in the native protein [38]. This position is equivalent to position 188 in AMPK as described by Ratner *et al*. [39]. Cys208 in contrast is located after the last β-sheet and in front of the last α-helix and could show substantially higher flexibility and (folding) movements that are disconnected to the CORE domain [38]. It is however still part of the CORE domain and thus also a reporter for global and highly coupled folding events as reported for folding studies with AMPK [39].

These labels are placed to probe for potential variations in folding of a stabilizing central folding nucleus that was postulated for other α/β proteins [40] (Flavodoxin, CheY and Cutinase). Comparison of position 197 versus 208 could indicate such variations, since the latter is already positioned at the end of the CORE region, just before the last secondary structure element.

**Figure 11:**
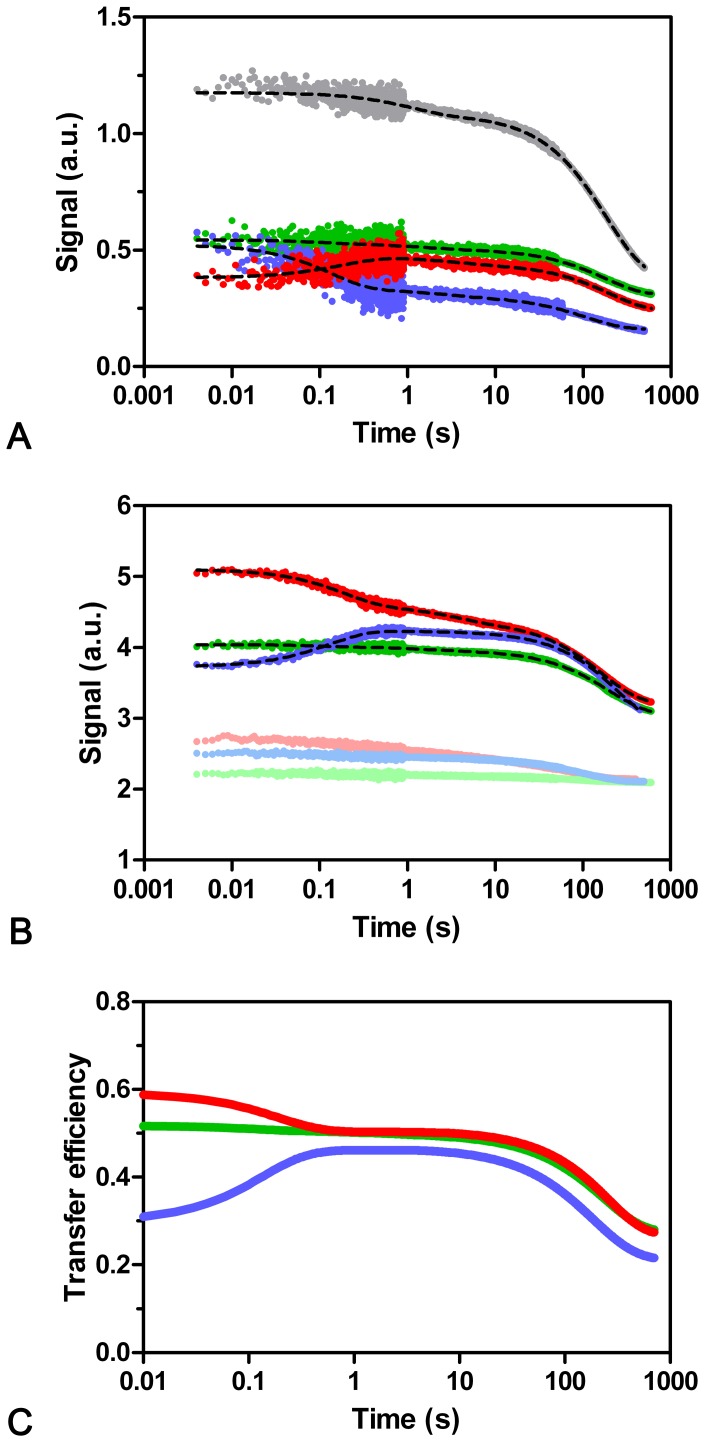
Refolding of labeled CMPK mutants. AEDANS attached at different positions to CMPK (see Fig. 1) serves as FRET acceptor with the single tryptophan as FRET donor. This leads to decrease in fluorescence of the (Trp) donor (a) and increase in fluorescence of (AEDANS) the acceptor (b) when the fluorophores approach upon folding. Excitation was performed at 296 nm. Gray: Trp signal of D+A− mutants; blue: *88; green: *197; red: *208. The light-colored traces in (b) correspond to the AEDANS-signal of D-A+ mutants. A double exponential is no longer sufficient to describe the data, and a triple exponential has to be used instead. The opposing data-courses within the first second illustrate the effect in energy transfer (increase in *88, decrease in *208 and no change in *197). The transfer efficiencies for each mutant based on donor quenching and sensitized acceptor-emission calculated from global fit results of single and double labeled mutants is displayed in (c). The major changes are linked to the burst, fast and slow phase, while the intermediate phase shows almost no change in transfer efficiency.

The corresponding refolding kinetics are shown in Fig. 11a/b and show individual characteristics for each variant. The *197 mutant shows only minor fluorescence changes in the range of the fast phase (<10 s), and the major signal change (>90%) is associated with the slow refolding phase with an observed rate constant λ_F3(RS)_ of approximately 0.006 s^−1^. The *88 mutant shows a strong increase in energy transfer within the first second (increase in AEDANS and decrease in Trp fluorescence) with a rate constant λ_F1(RS)_ of 5.4 s^−1^, and a decrease in fluorescence and energy transfer with a rate constant λ_F3(RS)_ of 0.005 s^−1^. With the *208 mutant the amount of energy transfer decreases in the fast process (rate constant λ_F1(RS)_ of 7.5 s^−1^) and like the other mutants followed by a further decrease with a rate constant λ_F3(RS)_ of 0.006 s^−1^.

These mutants in combination with energy transfer indicate that the refolding traces can no longer be sufficiently described with the sum of 2 exponentials. Instead, 3 exponentials have to be used, where a new intermediate phase with an average rate constant of 0.45 s^−1^ (*88∶0.29 s^−1^; *197∶0.41 s^−1^; *208∶0.66 s^−1^) is revealed. This could be connected to the intermediate rate constant λ_F2(IU)_ revealed in the interrupted unfolding reaction described above, that corresponded to a folding intermediate with *cis*-Pro configuration. In how far these two transitions correspond to the same folding intermediate is not clear, since both experiments start from different initial conditions. But since the magnitudes of amplitude change and rate constants are comparable, it might in both cases correspond to the same transition. Though far from being proven, the unfolded proteins with a *cis*-Pro configuration might as well burst to a folding intermediate that behaves similar to the one described in interrupted unfolding experiments.

The time course of energy transfer for each mutant is displayed in Fig. 11c (calculation see Methods). Within the burst phase, the energy transfer increases from values <0.1 in the unfolded state (verified with fluorescence decay experiments, data not shown) to 0.3 in *88, 0.5 in *197 or 0.6 in *208. Within the fast transition, the energy transfer changes to values of 0.4–0.5 for all mutants, and afterwards decreases to approximately 0.3, which was independently determined as the transfer efficiency value in the folded state (fluorescence decay, data not shown). The intermediate phase is not associated with significant changes in transfer efficiency.

## Discussion

### Folding Kinetics of CMPK

The folding properties of several NMP kinases that belong to the family of proteins with α/β topology (e.g. like Flavodoxin) were studied recently. The by far most extensively investigated member of this family is adenylate kinase from *Escherichia coli* (AMPK) where several groups made important contributions [37]–[39], [41], [42]. In addition, folding studies on UMP/CMP-kinase from *Dictyostelium discoideum* (UMPK) and studies of adenylate kinases from other sources also contributed to our current view on the folding properties of NMP kinases [4], [14], [25]. The comparison with folding properties of CMPK described here, another member of this protein family, provides interesting results in the context of protein folding properties in one family with highly similar 3D structure and yet pronounced variations in topology [6], [10], [40], [43]–[46].

### Kinetics of CMP-Kinase Folding

A scheme with the different time windows and kinetically observable intermediates of CMPK folding is shown in figure 9 It illustrates the 3 time regimes that could be resolved with apparent rate constants of folding (λ_F1(RS)_- λ_F3(RS)_) with 2, 0.2, 0.006 s^−1^, and unfolding (λ_U2(US)_) with 0.01 s^−1^.

Double jump experiments indicate that the intermediate refolding phase λ_F2(RS)_ is caused by a folding intermediate with Pro124 in *cis* configuration. For reasons of clarity and due to insufficient accumulation of this species, it was not included in the folding scheme but is presumably similar to λ_F2(IU)_, where the unfolded protein with *cis*-Pro124 could burst into the corresponding folding intermediate, folding to the native state with λ_F2(RS)_. The other two folding phases are likely caused by a folding pathway originating from intermediates with Pro124 in *trans*-configuration, where the slow process is influenced by proline isomerization. In the unfolding pathway, the slow process is connected to nearly complete loss of protein structure presumably including proline isomerization from *cis* to *trans*, while an additional unfolding step of 0.1 s^−1^ is spectroscopically silent.

This folding pathway as outlined here, is in general similar to the one described for AMPK [47] and also to the one described for UMPK [29], although for the latter folding intermediates could be assigned to be off-pathway. For both proteins, the slowest step was also assigned to *cis/trans* isomerization of a single *cis*-proline residue.

The kinetic phases of unfolding and refolding of CMPK as obtained with different methods (Including FRET) coincide within a narrow range for the wildtype protein as well as the generated mutants, respectively. This indicates concerted folding events, albeit not necessarily high cooperativity given the low m value and shallow slopes of the chevron plot [48]. Still protein folding of CMPK is not composed of totally concerted folding movements, since differences between individual subdomains of the protein structure can be observed, as indicated by refolding transitions of the individual mutants carrying an AEDANS-fluorophore. All three constructs show burst phases for the transfer efficiency (FRET signal) as well as changes associated with the slow refolding transition. For the *88 and *208 mutants, transfer efficiency changes are also associated with the fast phase. The middle phase shows no changes in transfer efficiency.

Interestingly, no change in the whole refolding transition can be observed for the direct excitation of AEDANS in the single-labeled *197 mutant. This suggests that either a stable folding core around Ala197 is generated within the burst phase of refolding, or that AEDANS at position 197 is totally solvent-exposed during all processes and does not encounter changes to its immediate surrounding.

A possible picture that emerges from these considerations is that a folding burst leads to formation of a central core region containing Ala197 and generation of secondary structure elements, while the kinetically detectable fast refolding-phase with Pro124 in *trans*-configuration leads to rearrangements in the “more flexible” regions including Trp31, Ser88 and Ser208. This would be consistent with NMR studies published by Waltz and co-workers [38], where AMPK shows a stable core with higher binding energy than peripheral protein regions, leading to separate folding events. Finally, the slow refolding transition, presumably including proline-isomerization, leads to major rearrangements of the protein structure in a slow folding process, since FRET transfer efficiency displays significant changes for all three mutant positions and 25% change of the overall amplitude in secondary structure occurs in this step. This is similar to the data from proteins with flavodoxin-like fold [43] where a molten globule state around the central α/β nucleus could be observed.

This folding pathway would also explain the observed tryptophan fluorescence intensities during refolding. In the unfolded conformation, Trp31 is solvent-exposed and quenched by solvent molecules. During the initial collapse it is transferred to a hydrophobic surrounding in the intermediate states I^t^_1_ and I^t^_2_ with reduced quenching and increased tryptophan fluorescence. Finally, the slow refolding transition leads to a decrease of fluorescence at 360 nm due to embedding of the tryptophan residue into the protein lattice and quenching by specific interaction especially with the protein backbone [49].

### Folding Kinetics of CMPK in Relation to Other NMP Kinases

When comparing these results to data from other NMPK’s, similar folding kinetics within the protein family can be observed, as well as unique differences that might arise from specific variations of the individual protein structures.

The unfolding/refolding scheme of CMPK is largely comparable to the one described for AMPK [47] with similar amounts of intermediates as well as comparable unfolding and refolding kinetic rate constants except for the slow unfolding process. Interestingly, it was shown [37] that loop 28–71 of AMPK forms early in the folding process, yet with a non-native distance between residues 58–86, indicating a potentially late positioning of the NMP-domain in the folding process, comparable to our results for CMPK. Also folding kinetics of UMPK [29] showed two refolding phases of major amplitude changes, but one structural unfolding transition that was independent of proline isomerization. This suggests that refolding of NMP kinases is indeed conserved between the individual members of this protein family, whereas unfolding of CMPK differs from the other proteins.

In comparison to other NMPKs characterized so far, it is evident that CMPK is thermodynamically more stable, but even more so kinetically. UMPK is half denatured in equilibrium at [urea]_1/2_ 2 M, AMPK at 2.1 M and CMPK at 3.1 M. The slowest step of unfolding occurs with 0.015 s^−1^ (at 4 M urea) for UMPK, 0.1 s^−1^ (at 1M GndCl) for AMPK but only 0.001 s^−1^ (at 4 M urea) for CMPK. Even more pronounced is the extrapolation of the slow unfolding phase from the chevron plots to no denaturant: here AMPK and UMPK unfold with 10^−2^–10^−3^ s^−1^ but CMPK with 10^−4^–10^−5^ s^−1^ [29], [41], [47] and thus two orders of magnitude slower.

The major structural differences between CMPK and the other two NMP kinases are a specific large insert of 40 amino acids as well as the unique positioning of the single *cis*-proline residue within the protein structure. As described by Briozzo *et. al.* [12] CMPK like other NMP kinases contains a central 5-fold parallel β-sheet but additionally contains an insert of 40 amino acid residues in the NMP-binding domain (Leu 63– Gln102), which is composed of a three-stranded antiparallel β-sheet, and two α-helices. This insert has a large interface with the central CORE domain that allows a gliding movement during the catalytic cycle [12]. This interface increases the surface between CORE and NMP domain roughly by a factor of two. It is therefore likely to act as a stabilizing component for the folded protein conformation, leading to the by two orders of magnitude decelerated unfolding kinetics relative to the other NMP kinases. Since this long NMP-insert is unique for CMPK, a different propensity of folding and unfolding intermediates along the folding funnel might be present, compared to other NMP kinases with higher flexibility of the LID- and NMP-domains. This could as well explain the different abundance of intermediate states especially observed for double jump experiments of CMPK versus UMPK [29]. Unfortunately a CMPK mutant lacking the long NMP insert (Δ Leu63-Gln102) could not be successfully purified to further validate this working hypothesis.

Next to the insert, the specific position of Pro124 located in a hinge-region between the CORE and NMP-domain in CMPK could well contribute to the decelerated unfolding kinetics. Nearly all NMPKs whose structures were determined to date contain one *cis* proline, and undergo substrate induced structural changes (induced fit) to reach the catalytically active state [26]. In the other NMP-kinases studied so far this single *cis*-proline residue is located in a flexible unstructured region within the CORE domain with no major predicted rearrangements upon conversion of *cis* to *trans* (see Fig. 2). In the case of CMPK, *cis*/*trans* isomerization however should disrupt a substantial amount of interaction of the NMP-domain (and its insert) with the CORE domain, leading to a higher kinetic barrier for unfolding.

The study of E.coli CMPK folding nicely shows how how only minor adjustments in topology within a protein family significantly impacts the folding landscape.

## Materials and Methods

### Cloning, Expression and Purification

The *cmpk-wildtype* gene was amplified from *E. coli* genomic DNA via PCR with the following primers: CMPK-sense: GATATTCCAT**ATGACGGCAATTGCCCCGG** **,** CMPK-anti: GAATGCTAGCTTATTAGTGGTGGTGGTGGTGGTG **TGCGAGAGCCAATTTCTG** **.** (Bold: gene; underlined: His-Tag) The fragment was cloned into a pET−24b(+) expression vector (Novagen, Madision, WI, USA) via NdeI and NheI restriction sites, which adds a non-cleavable N-terminal His-tag to the protein. The plasmid was transformed into *E. coli* BL21(DE3) cells and protein expression was induced by addition of 0.5 mM IPTG at an OD_600_ of 0.6. The cells were incubated at 20°C overnight, then harvested and resuspended in 50 mM Tris/HCl, pH 7.5, 300 mM KCl, 20 mM imidazole, pH 7.5 and 5 mM β-mercapto-ethanol (lysis buffer), containing 1 µM PefaBloc®, 1 mg/ml lysozyme and 5 µg/ml DNAseI. For cell lysis, the suspensions were shock-frozen in liquid nitrogen, thawed and then ultrasonicated for 5 minutes. Removal of cell debris and insoluble proteins was performed via ultracentrifugation for 60 minutes at 20,800 rcf and 4°C (eppendorf 5417 R centrifuge). The supernatant was applied to a gravity-flow nickel-nitrilo-triacetic-acid (Ni-NTA) agarose-column (Qiagen, Hilden, Germany) equilibrated in lysis buffer. After elution of the bound protein, 2.5 U alkaline phosphatase/ml initial pellet volume was added to remove remaining nucleotide and the sample was dialyzed against 2 l storage buffer overnight. After concentration using centrifugal filter devices (Millipore, molecular mass cut-off 10 kDa), the protein was applied to a gel filtration on a sephadex 75 26/60 gel filtration column (GE Healthcare, Fairfield, USA), equilibrated in 50 mM Tris/HCl, pH 7.5, 100 mM KCl and 2 mM DTE (storage buffer). CMPK was collected in the fractionated flow-through and concentrated to more than 8 mg/ml. Around 40 mg of protein per liter of culture was obtained with a purity of >95%, as judged by sodium dodecyl sulfate polyacrylamide gel electrophoresis (SDS-PAGE) with Coomassie staining. The correct mass of 25.6 kDa was confirmed by matrix-assisted laser desorption/ionization time-of-flight (MALDI-TOF) mass spectrometry (Shimadzu Europa GmbH, Germany). The activities of the created variants ranged from 88 to 196% wildtype-activity.

### Labeling with IAEDANS

The protein was applied to a NAP-10 column (GE Healthcare, Fairfield, USA) equilibrated in 50 mM Tris/HCl, pH 7.5 and 100 mM KCl to remove DTE from the buffer. 1 mM 5-[2-[(2-Iodo-1-oxoethyl)amino] ethylamino]-1-naphthalenesulfonic acid (IAEDANS) (Invitrogen, Darmstadt, Germany), 10-fold excess over protein and dissolved in the same buffer, was added dropwise to the protein solution. After 6 hours reaction time at 4°C and constant shaking, the reaction was terminated by applying the labeling solution to a NAP-10 column, equilibrated with DTE containing storage buffer. The labeled protein was dialyzed against 2 l storage buffer and concentrated to more than 7 mg/ml. Label efficiency was determined with MALDI-TOF measurements for all variants to be ≥94.5%.

### Equilibrium Unfolding Transitions

Urea-induced equilibrium denaturation of CMPK was carried out with freshly prepared stock solutions to reduce effects from reactive cyanate ions. Exact urea concentration of stock solutions was determined refractometrically as described by Warren and Gordon [50].

Equilibrium unfolding measurements were carried out with 5 µM CMPK in 50 mM Tris/HCl, pH 7.5, 100 mM KCl and 2 mM DTE. After several hours of equilibration the fluorescence-signal was recorded between 310 and 500 nm in steps of 1 nm at 25°C in a Fluoromax fluorometer system (Horiba Europe GmbH). For analysis, data was added up to slices of 10 nm. Fluorescence of the *88 mutants was recorded in a Varioskan Flash microtiterplate reader (Thermo scientific) between 306 and 600 nm in steps of 1 nm at 25°C. Far-UV CD measurements of the same samples were carried out with a Jasco J-810 spectropolarimeter (Jasco GmbH, Groß-Umstadt, Germany). Spectra between 210 and 250 nm with a resolution of 1 nm were recorded at 25°C with a cuvette of 0.1 cm path length and the band pass set to 1 nm.

The secondary plots were fitted according to a two state unfolding transition, using the equation described by Santoro and Bolen [51]:
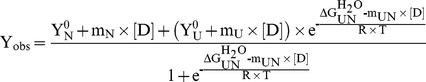
(1)Here Y_obs_ is the observed spectroscopic signal, while Y_N_^0^ and Y_U_^0^ are the spectroscopic signals of the native and the unfolded state. m_n_ and m_u_ are the denaturant dependent slopes of the signal in the native and unfolded state. 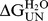
is the free energy of unfolding in water and m_UN_ displays its dependence on concentration of denaturant and is given in J mol^−1^ M^−1^. It also describes the exposure of amino acid residues to the solvent. R is the gas constant and T the temperature in Kelvin.

### Kinetic Measurements

Folding kinetics were measured with a BioLogic SFM 400 stopped-flow apparatus including a FC15 cuvette and a high density mixer. The mixing dead-time of the instrument was about 3 ms in the single jump and 60 ms in the double jump mode. The specific wavelength region for photomultiplier detection was defined by optical filters (tryptophan fluorescence: 360 nm band-pass filter, AEDANS fluorescence: 475 nm long-pass filter, both LOT, Darmstadt, Germany) upon excitation at 296(Trp or FRET) or 336 nm (AEDANS directly).

The final CMPK concentration of each measurement was 4 µM (5 µM for labeled mutants) in 50 mM Tris/HCl, pH 7.5, 100 mM KCl and 2 mM DTE at 25°C. For refolding experiments CMPK was incubated for 2 hours in buffer containing 6 M urea at 25°C before refolding was initiated by 10-fold dilution into buffer without urea. For unfolding experiments, CMPK was incubated for 2 hours in 0.6 M urea at 25°C, before unfolding was initiated by 10-fold dilution into buffer containing 6 M urea. Due to the higher complexity of the refolding transition, unfolding was analyzed only with the wildtype and the *88 CMPK variants, while refolding was analyzed with the wildtype and the *88, *197 and *208 variants. For CD data, the final concentration inside the cuvette was increased to 10 µM CMPK and the CD signal was recorded at 222 nm. Specifically multi-phase reactions were measured with different time windows that differ in the individual times signals could be sampled and thus S/N ratio.

For the D+A−(including the wildtype) and D−A+ variants, illumination at 296 nm resulted in specific excitation of the according fluorophore and was used to evaluate the specific fluorescence of tryptophan and AEDANS in the absence of FRET. Data obtained from the D-A+ variants was correlated to the data from excitation at 336 nm in order to exclude differences in the AEDANS-fluorescence depending on the excitation wavelength or energy transfer from other residues. Illumination at 296 nm of D+A+ lead to direct excitation of both fluorophores as well as modulation of the fluorescence signals by energy transfer, depending on the structural conditions of the protein.

For data analysis, multiple datasets were averaged. The kinetic traces were fitted to single-, double or triple-exponential equations using the software Prism4 (GraphPad).

### Determination of Energy Transfer Efficiency E and Average Apparent Distance <R(t)>

We determined the energy transfer efficiency from donor as well as acceptor fluorescence using the results from global fits to a 3-exponential function of a complete set of mutants ((D+A−), (D+A+) and (D−A+) of *88, *197 or *208). Transfer efficiency by quenching of donor fluorescence was determined by
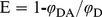
(2)where φ_D_ and φ_DA_ are the quantum yields for the (D+A−) and the (D+A+) variants, respectively. According to Fairclough and Cantor [52], transfer efficiency calculated from sensitized acceptor-emission was analyzed by



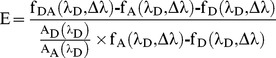
(3)Where f_i_(λ_D_, Δλ) is the fluorescence intensity of variant i in the interval Δλ for excitation at wavelength λ_D_. A_j_(λ_D_) is the absorption of fluorophore j at wavelength λ_D_. The results of both measurements were averaged.

### Fluorescence Lifetime

The donor fluorescence lifetime of all variants carrying Trp31 were analyzed with a PicoQuant PDL 800-B pulsed diode laser with a PLS 295 sub-nanosecond pulsed LED (spectral center at 295 nm, spectral width of 12 Å). For detection of donor fluorescence, a 350 nm band pass filter was inserted into the light path behind the sample chamber. All probes were equilibrated in 0.6 M and 6.0 M urea at a concentration of 10 µM. After data acquisition, datasets were fitted with the PicoQuant FluoFit software v. 4.2.9 using the built in exponential model reconvolution fit. All datasets could be fitted with a model of two exponential components. From these results, an apparent fluorescence lifetime was calculated by
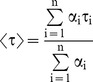
(4)with <τ> being the apparent fluorescence lifetime, τ_i_ being the lifetime and α_i_ the amplitude of the i-th component. By comparison of the apparent lifetimes for the labeled (<τ_DA_>) and unlabeled (<τ_D_>) variants, the average transfer efficiency E was determined with
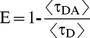
(5)


### Enzymatic Activity after Purification

Activity-assays were performed for all CMPK variants by coupling phosphorylation of CMP to CDP to a colorimetric assay [19]. The reaction buffer was composed of 100 mM Tris/HCl, pH 7.5, 80 mM KCl, 1.4 mM MgCl_2_, 2 mM DTE, 0.8 mM phosphoenolpyruvate, 0.4 mM NADH, 10 U/ml pyruvate kinase, 10 U/ml lactate dehydrogenase, 10 U/ml nucleoside di-phosphate kinase (NDK) and 1 mM ATP. NDK was added to the solution to achieve complete conversion of CDP to CTP [19]. The absorption signal at 340 nm was recorded with a Jasco V-650 UV/Vis Spectralphotometer at 25°C in order to follow depletion of NADH. The activity of different variants of 2 nM CMPK was determined with 0.5 mM CMP and approximately 0.01 mg/ml bovine serum albumin.
